# Occurrence and temporal distribution of extended-spectrum β-lactamase-producing *Escherichia coli* in clams from the Central Adriatic, Italy

**DOI:** 10.3389/fmicb.2023.1219008

**Published:** 2023-11-06

**Authors:** Francesca Leoni, Luca Sacchini, Silvia Pieralisi, Gabriele Angelico, Chiara Francesca Magistrali, Lucilla Cucco, Francesca Romana Massacci, Elisa Albini, Anna Duranti, Cesare Cammà, Barbara Secondini, Antonio Rinaldi, Francesca Barchiesi

**Affiliations:** ^1^Laboratorio Nazionale di Riferimento per il Controllo Delle Contaminazioni Batteriche dei Molluschi Bivalvi, Istituto Zooprofilattico Sperimentale Dell’Umbria e Delle Marche “Togo Rosati”, Ancona, Italy; ^2^Istituto Zooprofilattico Sperimentale Dell’Umbria e Delle Marche “Togo Rosati”, Perugia, Italy; ^3^National Reference Centre for Whole Genome Sequencing of Microbial Pathogens: Data-Base and Bioinformatics Analysis (GENPAT), Istituto Zooprofilattico Sperimentale Dell’Abruzzo e del Molise “G. Caporale”, Teramo, Italy; ^4^Centro di Referenza per il Controllo Microbiologico e Chimico dei Molluschi Bivalvi Vivi, Istituto Zooprofilattico Sperimentale Dell’Umbria e Delle Marche “Togo Rosati”, Ancona, Italy

**Keywords:** bivalves, *Escherichia coli*, ESBL, antimicrobial resistance, CTX-M, clam, *Escherichia ruysiae*, *Escherichia marmotae*

## Abstract

The spread of extended-spectrum β-lactamase (ESBL)-producing *Escherichia coli* is a major public health issue. Bivalves are filter-feeder animals capable of bioaccumulating the microorganisms present in water. This physiological characteristic makes them both good indicators of environmental contamination and possible carriers of pathogenic bacteria, including those resistant to antimicrobials. The aim of this study was to investigate the occurrence of ESBL-producing *E. coli* in clams (*n* = 308) collected from harvesting areas of the Central Adriatic Sea between 2018 and 2019. ESBL- /class C β-lactamase (AmpC)- producing *E. coli* and *Escherichia* spp. were isolated by streaking over the surface of MacConkey agar plates supplemented with cefotaxime enriched broths of the initial shellfish suspension. *E. coli* and *Escherichia* spp. resistant to cefotaxime were screened for ESBL production by using the double disk synergy test. Susceptibility to different antimicrobials and confirmation of ESBL-production were determined by the minimum inhibitory concentration (MIC) test. Isolates were further characterized by whole genome sequencing (WGS) and bioinformatic analysis of genomes with different tools. Overall, ESBL-producing *E. coli* were isolated from 3% of the samples. Of 13 ESBL- and ESBL−/AmpC-producing *Escherichia* spp. (*n* = 11 *E. coli*, *n* = 1 *E. marmotae*, *n* = 1 *E*. *ruysiae*) isolates, 13 were resistant to ampicillin and cefotaxime, 9 to sulfamethoxazole, 6 to tetracycline and nalidixic acid, 4 to trimethoprim, and 3 to ceftazidime, cefoxitin, ciprofloxacin, and chloramphenicol. Moreover, the majority (8/11) of the ESBL-producing *E. coli* isolates were multidrug-resistant. WGS showed that the isolates predominantly carried the *bla*_CTX-M-15_ gene (3/11) and *bla*_CTX-M-14_ and *bla*_CTX-M-1_ (2/11 each). The AmpC β-lactamase CMY-2 was found in two isolates. Phylogroup A was the most prevalent (5/11), followed by phylogroups D (4/11), F (1/11), and B2 (1/11). Ten different sequence types (STs) were identified. Occurrence at sampling sites ranged between 0 and 27%. To identify associations between the occurrence of ESBL-producing *E. coli* and *E. coli* levels, samples were divided into two groups, with *E. coli* at >230 MPN/100 g and *E. coli* at ≤230 MPN/100 g. ESBL-producing *E. coli* isolates were significantly more commonly recovered in samples with higher *E. coli* levels (14%) than in those with lower levels of *E. coli* (2%). Moreover, the majority (3/4) of the potentially pathogenic strains were isolated in samples with higher *E. coli* levels. These findings provided evidence for the bacterial indicator of fecal contamination, *E. coli*, as an index organism for ESBL-producing *E. coli* isolates in bivalves.

## Introduction

Antimicrobial resistance (AMR) is one of the most significant public health threats (World Health Organization, 2021), responsible for hundreds of thousands of estimated deaths annually worldwide (O’Neill, 2016).

Third/fourth/fifth-generation cephalosporins are the highest priority critically important antimicrobials (HPCIAs) in human medicine (World Health Organization, 2019), and Enterobacteriaceae producers of extended-spectrum β-lactamases (ESBLs) are on the critical-priority WHO list of antibiotic-resistant bacteria for research and development of new antibiotics (Tacconelli et al., 2018). An 8-fold increase in the intestinal carriage rate of ESBL *Escherichia coli* in the community has occurred globally over the past two decades (Bezabih et al., 2021), and the global and regional human intestinal carriage of ESBL *E. coli* is increasing in both community and healthcare settings (Bezabih et al., 2022). The spread of ESBL poses a serious threat to public health; therefore, it is important to investigate sources and transmission routes and encourage studies contributing to the “One-Health” approach.

Human- and animal-gut bacteria, including those resistant to antimicrobials, can reach marine environments through various routes (e.g., runoff from land, sewage systems, and feces from birds and wild animals), with the potential to contaminate seafood products. Bivalves are invertebrate filter-feeder animals capable of bioaccumulating microorganisms present in the surrounding waters. Thus, they are good indicators of environmental contamination and may act as possible carriers of bacteria derived from fecal pollution (Lee and Silk, 2013), including those that are resistant to antimicrobials (Albini et al., 2022).

In the European Union, regulation concerning the sanitary safety of live bivalve mollusks (Anonymous, 2004, 2019) stipulates that classified production areas shall be periodically monitored to check the microbiological quality of shellfish by using the bacteriological indicator of fecal contamination, *E. coli*. The latter is a genetically diverse species that comprises non-pathogenic gut commensals and strains responsible for intestinal and extra-intestinal disease. Enterotoxigenic *E. coli* (ETEC) strains are able to bind and colonize the intestinal epithelium and also produce various enterotoxins, of which heat-labile and heat-stable toxins and/or enteroaggregative heat-stable toxin 1 (EAST1) lead to diarrhea. Extra-intestinal pathogenic *E. coli* (ExPEC) are non-commensal *E. coli* isolates capable of causing extra-intestinal disease due to the possession of pathogenic virulence factors (Russo and Johnson, 2000). *Escherichia coli* isolates containing at least two genes coding virulence factors (*papA* and/or *papC*, *sfa*/*foc*, *afa*/d*raBC*, *kpsM II*, and *iutA*) are defined as ExPEC (Peirano et al., 2013).

Antimicrobial resistance monitoring programs in the EU are focused on terrestrial animals (Aerts et al., 2019). Studies on the occurrence of ESBL-producing *E. coli* in bivalves are limited. The prevalence of ESBL-producing *E. coli* has been investigated in retail bivalves in studies conducted in European or North African countries (Boss et al., 2016; Vu et al., 2018; Sola et al., 2022). In European studies, ESBL- or AmpC-producing *E. coli* isolates were not recovered in retail oysters sampled in Switzerland (Boss et al., 2016), and ESBL-producing Enterobacteriaceae were isolated in 20% of bivalve samples collected at retail in Berlin (Vu et al., 2018). In another study conducted in Tunisia, bivalves were sampled in unrelated markets in different regions, and ESBL-producing Enterobacterales (mostly *E. coli*) were cultured from 1.6% of clam pools (Sola et al., 2022). Other studies investigated the occurrence of ESBL-producing *E. coli* isolates in bivalves from production areas (Rees et al., 2015; Bueris et al., 2022). Hence, there are relatively few studies assessing the occurrence of ESBL-producing *E. coli* in bivalves from production areas over different seasons, and none of them were carried out in Italy. Furthermore, to our knowledge, previous studies have not investigated the relationship between levels of the bacterial indicator of fecal contamination, *E. coli*, and ESBL-producing *E. coli* presence in bivalve mollusks.

Clam is a major commercial species in Italy, and among EU countries, Italy is the main producer, accounting for 77% of farmed clams in the EU, reaching 24,452 tons in 2020 (European Commission, 2022). As clams are grown in coastal waters, they can also represent a sentinel species in determining AMR in the marine environment.

The aim of this study was to investigate the occurrence of ESBL-producing *E. coli* in clams collected from harvesting areas of the Central Adriatic Sea between 2018 and 2019 and study the correlation between ESBL-producing *E. coli* and levels of *E. coli*, the bacterial indicator of fecal contamination of bivalve mollusks. Moreover, EBSL- and ESBL-/AmpC-producing *E. coli* isolates from bivalves were characterized phenotypically, for susceptibility to antimicrobials, and genotypically, by whole-genome sequencing (WGS), to assign them to a serotype, phylogroup, sequence type (ST), and identify the presence of resistance and virulence genes and mutations that confer antimicrobial resistance.

## Materials and methods

### Sampling

A total of 308 samples of clams (*Venus gallina*), collected from 28 sampling sites of harvesting areas located along the coast of the region of Marche (Supplementary Appendixes 1, 2), were analyzed for the presence of *E. coli*-producing ESBL and/or class C β-lactamase (AmpC). Of these, 127 were from bivalve mollusk harvesting areas classified as B, which requires a post-harvest treatment before being placed on the market to meet *E. coli* health parameters. The remaining 181 samples were from areas classified as A.

Monitoring for the presence of *E. coli* ESBL/AmpC was performed approximately each month between 2018 and 2019 for the majority (25) of the areas.

Bivalve mollusks were externally cleaned with running potable water; then, the flesh and liquor of the bivalve mollusks were aseptically collected, diluted, homogenized, and further diluted in a 0.1% sterile peptone water to achieve a final suspension of 1:10. Subsequent decimal dilutions were prepared in a 0.1% sterile peptone solution. *E. coli* enumeration on bivalve mollusks was performed by a most probable number (MPN) method according to ISO 16649-3 (Anonymous, 2015).

ESBL-/AmpC-producing *E. coli* were isolated by streaking over the surface of MacConkey (MC) agar plates supplemented with 1 μg/mL of cefotaxime (Sigma Aldrich-Merck KGaA, Darmstadt, Germany) enriched broths of the initial shellfish suspension in double-strength mineral-modified glutamate (MMGB) broth from the *E. coli* enumeration method. Inoculated MC agar plates with cefotaxime were incubated at 37° C ± 1° C for 24 h ± 2 h in aerobic conditions. Two colonies showing typical characteristics of *E. coli* were randomly selected from each sample and isolated in trypticase soy agar (TSA, Biolife, Italy).

### *Escherichia coli* identification

Presumptive colonies of *E. coli* were analyzed by PCR for the *uidA* gene with primers uidA-277F and uidA-934R^1^ and by matrix-assisted laser desorption ionization–time-of-flight mass spectrometry (MALDI-TOF MS Biotyper, Bruker Daltonics) analysis.

### Antimicrobial susceptibility testing and ESBL/AmpC phenotype

For the isolates identified as *E. coli*, disk diffusion susceptibility tests (EUCAST, 2017a) were conducted for nine antibiotics (Supplementary Appendix 2). Inhibition diameter sizes were interpreted by using the EUCAST breakpoint tables (EUCAST, 2018), except for nalidixic acid and tetracycline, for which CLSI breakpoint values were used (CLSI, 2019).

*E. coli* isolates resistant to cefotaxime were screened for ESBL production by using the double disk synergy test (DDST) (EUCAST, 2017b).

For each sample, according to antimicrobial resistance screening results, one *E. coli* isolate was selected and further investigated for the determination of the minimum inhibitory concentration (MIC) for different antimicrobial classes and for the confirmation of ESBL production. If differences were observed in the antimicrobial susceptibility profiles of *E. coli* isolates from the same sample, each isolate underwent MIC tests. MIC tests were performed with Sensititre EU Surveillance *Salmonella*/*E. coli* EUVSEC Plates and Sensititre EU Surveillance ESBL EUVSEC2 Plates (Thermo Fisher Scientific), according to the Thermo Scientific Sensititre Plate Guide for Antimicrobial Susceptibility Testing (Thermo Fisher Scientific).

Clinical breakpoints provided by the Clinical and Laboratory Standards Institute (CLSI, 2021) were used for the interpretation of MICs (S: susceptible, I: intermediate, SDD: susceptible-dose dependent, and R: resistant) of the following antimicrobials: ampicillin (AMP), cefepime (FEP), cefotaxime (FOT), cefoxitin (FOX), ceftazidime (TAZ), ertapenem (ETP), imipenem (IMI), meropenem (MER), colistin (COL), gentamicin (GEN), ciprofloxacin (CIP), trimethoprim (TMP), tetracycline (TET), chloramphenicol (CHL), sulfamethoxazole (SXT), and nalidixic acid (NAL). For tigecycline (TGC) and temocillin (TRM), for which no clinical breakpoints were available from CLSI, EUCAST clinical breakpoint tables (EUCAST, 2023) were used for MIC interpretation (S: susceptible and R: resistant). In the case of azithromycin (AZI), for which no clinical breakpoint was defined, the epidemiological cutoff (ECOFF) value of 16 mg/L (EUCAST, 2023) was used for the classification of *E. coli* isolates as susceptible/non-susceptible. Isolates were considered ESBL if ≥8-fold reduction was observed in the MIC of any of the cephalosporins (cefotaxime or ceftazidime) combined with clavulanic acid compared with the MIC of that cephalosporin alone (EUCAST, 2017b). Isolates resistant to cefoxitin and cefepime, negative to the synergy test, were characterized as ESBL based on genetic characterization.

Multidrug resistance (MDR) was considered when isolates were resistant to three or more antimicrobial classes (Magiorakos et al., 2012).

### DNA extraction and whole-genome sequencing

Genomic DNAs were extracted from 1 mL of logarithmic phase broth cultures from pure *E. coli* cultures by using the QIAamp DNA Mini Kit (Qiagen Inc., Hilden, Germany) following the manufacturer’s protocol for Gram-negative bacterial organisms. DNA was quantified with the Qubit fluorometer (QubitTM DNA HS Assay, Life Technologies, Thermo Fisher Scientific Inc.). DNA libraries were prepared by using the Nextera DNA Flex Library Prep Kit (Illumina Inc., San Diego, CA), according to the manufacturer’s manual, loaded onto NextSeq 500/550 Mid Output Reagent Cartridge v2, 300 cycles kit (Illumina Inc., San Diego, CA) and then sequenced on an Illumina NextSeq 500 platform, to generate 150 bp paired-end reads.

### Sequence analysis

Raw data were checked for quality, trimmed using Trimmomatic v0.36 (Bolger et al., 2014), and assembled using SPAdes genome assembler v3.11.1 (Bankevich et al., 2012). Quality checks of raw data and assembled genomes are reported in the Supplementary Appendix 3, 4.

The assembled genomes were analyzed by online tools available at the Center for Genomic Epidemiology (CGE), Technical University of Denmark.^2^ Briefly, the FASTA files were analyzed using the following CGE databases: ResFinder (v.4.1) for antimicrobial resistance genes (ARGs) and chromosomal point mutations associated with resistance (Camacho et al., 2009; Zankari et al., 2017; Bortolaia et al., 2020), multilocus sequence typing (MLST v.2.0.9) for defining the ST (Lemee et al., 2004; Bartual et al., 2005; Wirth et al., 2006; Jaureguy et al., 2008; Camacho et al., 2009; Griffiths et al., 2010; Larsen et al., 2012), PlasmidFinder (2.0.1) for plasmid replicons (Camacho et al., 2009; Carattoli et al., 2014), VirulenceFinder (2.0.3) for virulence determinants (Camacho et al., 2009; Joensen et al., 2015; Malberg Tetzschner et al., 2020), and SeroTypeFinder (2.0) for serotyping (Joensen et al., 2015). Ribosomal multilocus sequence typing (rMLST, last updated 13 September 2022) at the Public databases for molecular typing and microbial genome diversity (PubMLST) was used for species identification (Jolley et al., 2012).

The presence of chromosomal mutations was evaluated based on the criteria that one single chromosomal mutation in the *gyrA* gene confers low-level resistance to quinolones, and several mutations in DNA gyrase genes (*gyrA* and *gyrB*) and topoisomerase IV genes (*parC* and *parE*) are required to increase the level of quinolone resistance in Enterobacteriaceae (Correia et al., 2017). ARG or plasmid replicons were considered present if length coverage and identity to the reference sequence were 100% and ≥ 95%, respectively. Virulence genes were considered present if length coverage and identity to the reference sequence were 100% and ≥ 90%, respectively.

*Escherichia coli* phylogroup and *Escherichia* clade assignment was performed *in silico* (Beghain et al., 2018) with ClermonTyping 21.03.^3^

To gain insight into the chromosomal or plasmid location of ESBL- /AmpC-encoding genes, assembled genomes were analyzed by MOB-suite (Robertson and Nash, 2018; v.3.0.3) to predict plasmid- and chromosome-derived sequences. Contigs harboring ESBL/AmpC-encoding genes were analyzed with ResFinder (v.4.1), PlasmidFinder (v2.0.1), and MobileElementFinder (v1.0.3; Johansson et al., 2021).

The raw sequencing data have been submitted to NCBI’s Sequence Read Archive (SRA) repository (BioProject: PRJNA882336, BioSample accessions SAMN30930934 to SAMN30930946).

### Statistical analysis

Quantitative *E. coli* results were divided into two groups based on the level of fecal contamination (*E. coli* ≤ 230 MPN/100 g and *E. coli* > 230 MPN/100 g). Statistical analysis was performed with Fisher’s test (Stata 16.1®), and values of *p* < 0.05 were considered statistically significant.

To study the seasonality of ESBL *E. coli* in clams, samples were categorized as summer (21st of June to 22nd of September)–autumn (23rd of September to 20th of December), and winter (21st of December to 20th of March)–spring (21st of March to 20th of June), according to the season of collection.

## Results

### Occurrence of ESBL-and ESBL/AmpC-producing *Escherichia coli* and other ESBL-producing *Escherichia* species isolates in clam samples

Overall, ESBL-producing *E. coli* isolates were cultured from 10 (3%, C.I.: 2–6%) of the 308 clam samples collected between July 2018 and November 2019 from the 28 sampling points. Of these, six (2%, C.I.: 0.7–4%) and three (1%, C.I.: 0.2–3%) samples harbored ESBL- or ESBL-/AmpC-producing *E. coli* isolates, respectively, while both types of isolates were recovered from one sample. Of note, other ESBL-producing *Escherichia* species were isolated, specifically *E*. *ruysiae* from one sample, and *E. marmotae*, from another sample, which also harbored an isolate of ESBL-/AmpC-producing *E. coli*. The latter species were presumably identified as *E. coli*, by PCR for the *uidA* gene and MALDI-TOF, and subsequently as *E. marmotae* and *E*. *ruysiae* by rMLST (100% with 53 exact matches) of WGS data.

### Antimicrobial resistance phenotype of ESBL- and ESBL-/AmpC-producing *Escherichia coli* and other ESBL-producing *Escherichia* spp.

Distribution of MIC values among the 13 *Escherichia* spp. isolates is reported in Table 1. Overall, all 13 ESBL- and ESBL-/AmpC-producing *Escherichia* spp. (*n* = 11 *E. coli*, *n* = 1 *E. marmotae*, *n* = 1 *E*. *ruysiae*) isolates showed resistance to ampicillin and cefotaxime, while 2 and 3 isolates were resistant to cefepime and ceftazidime, respectively, and 4 isolates had intermediate susceptibility to ceftazidime. Resistance and intermediate susceptibility to cefoxitin were found in 3 and 1 of the 13 isolates, respectively. Moreover, resistance to non-beta-lactam antibiotics was also observed to nalidixic acid (6/13), tetracycline (6/13), chloramphenicol (3/13), trimethoprim (4/13), sulfamethoxazole (9/13), gentamicin (2/13), and azithromycin (2/13). Of note, resistance to ciprofloxacin was found in three of the isolates. All (13 out of 13) isolates showed susceptibility to carbapenems (ertapenem, imipenem, and meropenem). Additionally, all isolates were susceptible to tigecycline, colistin, and temocillin. The majority (8 out of 11) of the ESBL- or ESBL-/AmpC-producing *E. coli* isolates were MDR (Table 2). The ESBL-producing *E. marmotae* was resistant to ampicillin and cefotaxime (Table 2), whereas the *E*. *ruysiae* was resistant to ampicillin, cefotaxime, and sulfamethoxazole and intermediate- and susceptible-dose dependent to ceftazidime and cefoperazone (Table 2).

**Table 1:** Distribution of MIC (minimum inhibitory concentration) values among the 13 ESBL- or ESBL-/AmpC-producing *Escherichia coli* (11 isolates) and ESBL-producing *E. marmotae* (1 isolate) and *E*. *ruysiae* (1 isolate) from clams.

Antibiotic molecule	0.015	0.03	0.06	0.12	0.25	0.5	1	2	4	8	16	32	64	128	256	512	1,024
Ampicillin													13 (100)				
Cefoxitin								1 (8)	3 (23)	5 (38)	1 (8)	2 (15)	1 (8)				
Ceftazidime							2 (15)	4 (31)		4 (31)	2(15)	1 (8)					
Cefotaxime									1 (8)	2 (15)	5 (38)	1 (8)	4 (31)				
Cefepime						1 (8)	1 (8)	2 (15)	4 (31)	3 (23)	1 (8)	1 (8)					
Tetracycline								7 (54)					6 (46)				
Tigecycline					11 (85)	2 (15)											
Meropenem		13 (100)															
Imipenem				6 (46)	7 (54)												
Ertapenem	6 (46)	4 (31)	2 (15)		1 (8)												
Ciprofloxacin	3 (23)	1 (8)		1 (8)	5 (38)					3 (23)							
Nalidixic acid									5 (38)	1 (8)	1 (8)		2 (15)	4 (31)			
Colistin							9 (69)	4 (31)									
Trimethoprim					6 (46)	3 (23)						4 (31)					
Chloramphenicol										10 (77)			1 (8)	2 (15)			
Gentamicin						4 (31)	7 (54)				1 (8)	1 (8)					
Sulfamethoxazole										4 (31)							9 (69)
Azithromycin								1 (8)	5 (38)	4 (31)	1 (8)		2 (15)				
Temocillin									1 (8)	9 (69)	3 (23)						

Percentages are shown in brackets. The shaded areas show the range of values tested for each antibiotic.

**Table 2:** Phenotypic antimicrobial resistance (AMR) profile, resistance genes, and gene mutations for AMR of ESBL- and ESBL-/AmpC-producing *Escherichia* spp. isolates from clams.

Isolate No.	β-lactamase profile	Phenotypic AMR	Resistance genes and/or mutations							
			β-lactams	Quinolone and Fluoroquinolone	Tetracycline	Aminoglycoside	Sulf	Phenicol and trimethoprim	Spec	MLS
AN2	ESBL	AMP AZI (NS) FOT NAL TET SMX TMP (MDR)	***bla***_**CTX-M-27**_	*gyrA* (p.S83L)	***tet(A)***	***aph (3′′)-Ib aph(6)-Id aadA5***	***sul1 sul2***	***dfrA17***	***aadA5***	***mph(A)***
AN9	ESBL	AMP FOT FEP (SSD) TAZ (I) SMX	***bla***_**CTX-M-15**_							
AN1	ESBL	AMP FOT TAZ CHL NAL TET SMX (MDR)	***bla***_**SHV12**_	*gyrA* (p.D87N)	*tet(A)* ***tet(B)***	***aadA1*** *aadA2b*	***sul3***	*cmlA1*	***aadA1*** *aadA2b*	
AN 5	ESBL	AMP FOT	***bla***_**CTX-M-1**_							
AN6	ESBL/AmpC	AMP FOT TAZ NAL TET FOX CIP (MDR)	***bla***_**CMY-2**_ ***bla***_**OXA-1**_ *bla*_TEM-126_*/bla*_TEM-106_*bla*_TEM-1B_	***aac(6′)-Ib-cr*** *parC (p.S80I)* gyrA (p.D87N) *gyrA* (p.S83L)	***tet(A)***	***aac(6′)-Ib-cr***				
AN8	ESBL	AMP AZI (NS) FOT FEP (SSD)	***bla***_**CTX-M-14**_	*aac(6′)-Ib-cr*		*aac(6′)-Ib-cr **aac(6′)-Ib3***		*cmlA1*		***mph(A)***
AN3	ESBL	AMP FOT TMP TET SMX FEP (SSD) (MDR)	***bla***_**CTX-M-1**_ ***bla***_**TEM-1B**_	***qnrS1***	***tet(A)***	***aph(3″)-Ib aph(6)-Id*** *aadA2b*	***sul2***	***dfrA5***	*aadA2b*	***mph(A)***
AN4	ESBL/AmpC	AMP FOT TAZ (I), NAL FOX FEP (SSD)	***bla***_**CTX-M-14**_ ***bla***_**CMY-2**_ ***bla***_**TEM-1B**_	*gyrA* (p.S83L)						
AN13	ESBL/AmpC*	AMP FOT SMX FOX FEP (SSD) (MDR)	*bla*_CTX-M-1_				***sul2***			
AN7	ESBL	AMP FOT CIP TAZ (I) CHL NAL TMP TET SMX FEP (SSD) (MDR)	***bla***_**CTX-M-55**_ ***bla***_**TEM-1B**_	*gyrA* (p.D87Y) g*yrA* (p.S83L) *parE* (p.S458A) *parC* (p.S80I)	*tet(A)*	***aph(3′)-Ia** aph(3″)-Ib* ***aph(6)-Id***	***sul2***	*catA2* ***dfrA14***		
AN10	ESBL	AMP FOT TAZ (I) FEP SMX	***bla***_**CTX-M-15**_ ***bla***_**TEM-**__**35**_	*gyrA* (p.S83A)						
AN11	ESBL/AmpC**	AMP FOT CIP TAZ NAL GEN SMX FOX (I) FEP (MDR)	***bla***_**CTX-M-15**_ ***bla***_**OXA-1**_ ***bla***_**TEM-**__**1B**_	***aac(6′)-Ib-cr*** *gyrA* (p.D87N) *gyrA* (p.S83L) *parC* (p.S80I) *parC* (p.E84V) *parE* (p.I529L)		***aac(6′)-Ib-cr*** *aac(3)-IIa*				
AN12	ESBL	AMP FOT CHL TMP TET GEN SMX FEP (SSD) (MDR)	***bla***_**CTX-**__**M-15**_ ***bla***_**TEM-1B**_	***qnrS1***	*tet(M) tet(A)*	***aph(3′)-Ia aph(3′′)-Ib*** *aph(6)-Id **aadA1** aac(3)-IId*	***sul2 sul3***	*cmlA1 **dfrA14 dfrA12***	***aadA1***	***lnu(F)***

*Mutations in the ampC-promoter: ampC-promoter: p.L9R, ampC-promoter:p.R8C, ampC-promoter:p.R11Q. **Mutation in the ampC-promoter: ampC-promoter:g.-28G > A. Genes indicated in black and bold were identified with 100% length coverage and 100% sequence identity. Genes indicated in black were identified with 100% length coverage and identity ≥ 95%. In brackets: SDD, susceptible dose-dependent; I, intermediate; NS, non-susceptible; Sulf, Sulfonamide; Spec, spectinomycin; MLS, macrolides–lincosamides–streptogramins.

### Genomic analysis of the ESBL- and ESBL-/AmpC-producing *Escherichia coli*

Among the 11 sequenced *E. coli* isolates, ARGs were detected for beta-lactams (*n* = 11), fluoroquinolones (*n* = 5), tetracyclines (*n* = 6), aminoglycosides (*n* = 8), sulphonamides (*n* = 6), phenicols (*n* = 4), trimethoprim (*n* = 4), spectinomycin (*n* = 4), macrolides (*n* = 3), and lincosamide (*n* = 1; Table 2).

ESBLs were encoded in 3 out of 11 *E. coli* isolates from clams by the *bla*_CTX-M-15_ gene; other common CTX-M variants were *bla*_CTX-M-14_ and *bla*_CTX-M-1_ (2 out of 11 isolates each), whereas *bla*_CTX-M27_ and *bla*_CTX-M55_ were each present in 1 of the 11 isolates (Table 2). Other ESBL-producing genes found in the *E. coli* isolates from clams were the *bla*_SHV12_ gene (1 out of 11 isolates) and one *bla*_TEM_ gene that had a sequence identity of 99.8% to *bla*_TEM-106_ (859/861 bp) and *bla*_TEM-126_ (859/861 bp), respectively_._ The combination of an ESBL (CTX-M-14 or a TEM enzyme with a gene sequence identity of 99.8% to *bla*_TEM-106_ and *bla*_TEM-126_) with a plasmidic AmpC β-lactamase (CMY-2) was found in two out of four of the isolates (Table 2). The remaining AmpC-producing *E. coli* isolates had mutations in the AmpC promoter (p.L9R, p.R8C, and p.R11Q; g.-28G > A, Table 2). The identified fluoroquinolone resistance genes were *aac(6′)-Ib-cr* and *qnrS1* in three and two isolates, respectively. Moreover, seven isolates had at least one point mutation known to mediate quinolone resistances in the chromosomal *gyrA*, while three isolates also possessed at least one additional mutation in the genes *parC*/*parE*. Tetracycline resistance genes *tetA*, *tetB*, and *tetM* were found in 6, 1, and 1 of the 11 isolates, respectively.

By the MOB-suite analysis of assembled genomes, 9 of the 11 contigs harboring ESBL-encoding genes and the two contigs harboring AmpC-encoding genes were classified as plasmid-derived sequences (Table 3; Supplementary Appendix 5). The presence of mobile elements and other antibiotic resistance genes in the same contigs harboring ESBL-/AmpC-encoding genes was also investigated and is reported in Table 3.

**Table 3:** Predicted genomic location (plasmid or chromosome) of ESBL- and AmpC-encoding genes using the MOB-Suite and presence of mobile genetic elements (MGEs) and other antimicrobial resistance genes in the same contig.

Isolate No.	ESBL-/AmpC-genes	ESBL/AmpC Contig ID and length	ESBL-/AmpC-gene position in Contig	MOB-Suite Contig classification	MGE in Contig*	Other genes
AN1	*bla*_SHV12_	NODE 108; 3,167 bp	2157.3017	Plasmid		
AN2	*bla*_CTX-M-27_	NODE 61; 1,513 bp	248.1123	Plasmid		
AN3	*bla*_CTX-M-1_	NODE 42; 3,843 bp	2598.3473	Plasmid		
AN4	*bla*_CTX-M-14_	NODE 149; 1,558 bp	564.1439	Plasmid		
	*bla*_CMY-2_	NODE 84; 9,387 bp	6989.8134	Plasmid	ISEc9	
AN 5	*bla*_CTX-M-1_	NODE 15; 101,323 bp	84738.85613	Chromosome	ISEc9	
AN6	*bla*_CMY-2_	NODE 44; 32,775 bp	17810.18955	Plasmid	ISEc9, IncI1	
	*bla*_TEM-126/_ *bla*_TEM-106_^**^	NODE 52; 19,233 bp	14392.15252	Plasmid	Tn2, IncX1	
AN7	*bla*_CTX-M-55_	NODE 1; 567,011 bp	152290.153165	Chromosome		
AN8	*bla*_CTX-M-14_	NODE 39; 13,443 bp	11952.12827	Plasmid	IS6100	*mphA*, *cmlA1, aac(6′)-Ib-cr aac(6′)-Ib3*
AN9	*bla*_CTX-M-15_	NODE 33; 5,012 bp	1898.2773	Plasmid	ISEc9	
AN10	*bla*_CTX-M-15_	NODE 9; 166,240	3287.4162	Chromosome	ISEc9	
AN11	*bla*_CTX-M-15_	NODE 43; 4,019 bp	432.1307	Plasmid		
AN12	*bla*_CTX_-_M-15_	NODE 69; 14,059 bp	1920.2795	Plasmid		
AN13	*bla*_CTX-M-1_	NODE 125; 4,978 bp	1226.2101	Plasmid		

*ISEc9: 100% (1656/1656 bp) sequence identity to GenBank accession number AJ242809; Tn2: 99.8% (4949/4950 bp) sequence identity to GenBank accession number HM749967; IncI1: 100% sequence identity to GenBank accession number AP005147; IncX1: 98.4% sequence identity to GenBank accession number EU370913; IS6100: 100% (880/880 bp) sequence identity to GenBank accession number X53635. **bla_TEM_ gene with a sequence identity of 99.8% to *bla*_TEM-106_ (859/861 bp) and *bla*_TEM-126_ (859/861 bp).

ESBL- and ESBL/AmpC-producing *E. coli* strains were diverse in serotype and fimbriae (Table 4). Most (9 out of 11) of the *E. coli* isolates were included in 6 different clonal complexes (Table 4); of these, CC10 and CC69 were present in 3 and 2 of the 11 ESBL and ESBL/AmpC *E. coli* isolates, respectively. Notably, the pandemic extra-intestinal pathogenic *E. coli* (ExPEC) ST131 clone (clonal complex CC131) was detected. Four phylogenetic groups (Table 5) were identified, of which phylogroup A was the most prevalent (45%), followed by phylogroup D (36%).

**Table 4:** Phylogroup, sequence type (ST), clonal complex (CC), replicon type, serotype, and virulence genes of ESBL- and ESBL-/AmpC-producing *Escherichia* spp. isolates from clams.

Isolate No.	β-lactamase profile	Phylogroup	ST No. (CC No.)	Replicon Type	Serotype	Virulence genes
AN2	ESBL	D	7401 (CC 69)	**IncFIA IncX4 IncFII(pRSB107)** IncFIB(AP001918) IncI2(Delta)	**H18**:O15	***chuA eilA fyuA gad irp*2 *iss*** *terC **ipfA ompT kpsE** kpsMII*
AN9	ESBL	Clade III	3568	**IncI1-I(Alpha) IncI2** IncFII(pHN7A8)	H56:*O36*	***astA iss traT*** *ompT sitA chuA gad terC kpsE kpsMII_K5*
AN1	ESBL	A	398 (CC 398) ETEC	IncFIB(AP001918) IncFII IncX1**IncI1-I(Gamma)**	**H20**:O8	***astA cmA gad hlyF iss iroN sitA traT*** *ompT terC*
AN 5	ESBL	V	14425	IncFIB (AP001918) IncFII(29)	H56:*O103*	***astA*** *hra traT chuA terC*
AN6	ESBL/AmpC	A	167 (CC 10)	**IncI1-I(Alpha)** Col156 IncFIB IncFIA (AP001918) IncFII IncX1 IncX4 p0111	H9:**O101**	***irp*2** ***cib*** ***celb*** ***fyuA*** ***gad*** ***iucC*** ***iutA*** ***senB*** ***sitA*** ***traT*** *terC* *capU* *iss*
AN8	ESBL	A	10 (CC 10)	**IncFII** Col156	H9:**O9a**	***irp*2 *terC*** *cea* *fyuA* *iss*
AN3	ESBL	D	69 (CC 69) ExPEC	**IncFII** IncFIB (AP001918)	**H18**O15	***air chuA cia cvaC eilA etsC fyuA gad hlyF iroN irp*2 *iss iutA kpsE lpfA*** *terC* ***kpsMII_K*5 *mchF*** o***mpT sitA traT*** *iha iucC*
AN4	ESBL/AmpC	A	10 (CC 10)	**Col(KPHS6)** Col156 IncB/O/K/Z IncFII IncFII(pHN7A8) IncI2 (Delta)	H10:**O29**	***celb*** ***fyuA*** ***irp*2** ***iucC*** ***iutA*** ***mcbA*** ***mchC*** ***terC*** ***traT*** *mchF sigA*
AN13	ESBL/AmpC	D	1299	IncFIB(AP001918) IncFII(pCoo) IncFII(pSE11) IncX1	H14:O175	***astA*** *chuA gad hra terC*
AN7	ESBL	F	457 ExPEC	IncFIB(AP001918)	O11	***cea chuA cma iss iucC iutA kpsMII ompT sitA traT yfcV*** *eilA gad hra terC kpsE lpfA papA papC*
AN10	ESBL	D	38 (CC 38)	Col156 IncFIB(AP001918) IncFII	**H18**:O86	***chuA fyuA irp2 iss kpsE senB sitA*** *eilA hra terC*
AN11	ESBL/AmpC	B2	131 (CC 131) ExPEC	IncFIB(AP001918) IncFII IncFIA	**H4**:O25	***chuA cnf1 fyuA hrA ihA irp*2 *iss iucC iutA kpsE kpsMII_K5 ompT papC sat sitA traT yfcV*** *terC gad*
AN12	ESBL	A	46 (CC 46)	IncFIB (AP001918) IncFIB(H89-PhagePlasmid)	H4:O8	***traT*** *iss terC*

Plasmid replicons were considered present if length coverage and identity to the reference sequence were 100% and ≥ 95%, respectively. Virulence genes were reported if length coverage and identity to the reference sequence were 100% and ≥ 90%, respectively. Genes indicated in black were identified with 100% coverage length and identity ≥ 95% for serotype. Genes indicated in black and bold were identified with 100% length coverage and 100% sequence identity. ExPEC, Extra-intestinal pathogenic *Escherichia coli*; ETEC, Enterotoxigenic *E. coli*.

**Table 5:** Phylogroups of 11 isolates of *E. coli* producers of ESBL or ESBL/AmpC from clam samples.

Phylogroup	ESBL (%)	AmpC (%)	ESBL/AmpC (%)	No. of isolates (%)
A	3 (43%)	0	2 (50%)	5 (45%)
B2	0 (0%)	0	1 (25%)	1 (9%)
D	3 (43%)	0	1 (25%)	4 (36%)
F	1 (14%)	0	0	1 (9%)
Total No. of isolates	7	0	4	11

Most strains harbored a broad virulence repertoire; moreover, in 3 out of 11 of the ESBL- and EBSL-/AmpC-producing *E. coli* isolates, at least two genes encoding for ExPEC virulence factors (*papA* and/or *papC*, *kpsM II*, *iutA*, *afa*/*draBC*, and *sfa*/*foc*) were identified (Table 4).

### Genomic analysis of the ESBL-producing *Escherichia* spp.

Among the 13 sequenced *Escherichia* spp., two isolates of ESBL producers were identified by rMLST (100% with 53 exact matches each) as *E. marmotae* and *E*. *ruysiae* and assigned by phylogroup analysis to clades V and III, respectively. The *E. marmotae* strain harbored the *astA* gene for the heat-stable enterotoxin 1 and other virulence genes (Table 4), whereas the ESBL was encoded by the *bla*_CTX-M-1_ (Table 2). The latter was predicted to be located in a chromosome-derived sequence (Table 3, AN5). A new ST (14425) was identified in the *E. marmotae* strain (Table 4). The *E*. *ruysiae* strain was assigned to ST 3568 by MLST analysis. Several putative virulence genes were predicted from the genome sequence of the *E*. *ruysiae* strain, including the enterotoxin *astA* gene (Table 4). The ESBL was encoded by the *bla*_CTX-M-15_ gene (Table 2), which was located in a contig classified as a plasmid-derived sequence (Table 3, AN9).

### Occurrence of ESBL- and ESBL-/AmpC-producing *Escherichia coli* isolates and seasonality at sampling points

**Figure 1:**
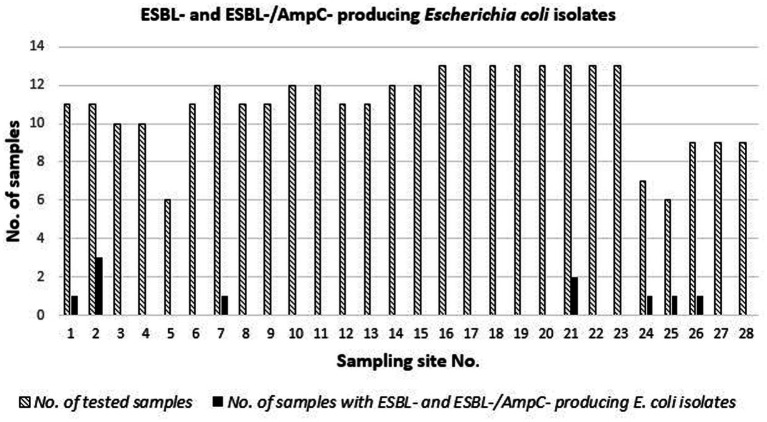
Occurence of ESBL-producing *E. coli* isolotes at 28 sampling sites of the Central Adriatic in 308 samples of clams collected between July 2018 and November 2019.

Over the studied period, the prevalence of ESBL- (including ESBL- and AmpC-)-producing *E. coli* isolates (Figure 1) ranged from 0% (no isolation) at 21 (75%) of the 28 sampling sites to 27% (3 out of 11 samples) in an area that was not suitable for the direct human consumption of bivalve mollusks.

Considering the seasonality, ESBL-producing *E. coli* were isolated in 3 of 85 (4%, C.I.95 1–10%), 3 of 62 (5%, C.I.95 1–14%), and 4 of 54 (7%, C.I.95 2–18%) of the samples collected in autumn, winter, and spring, respectively. Furthermore, ESBL-producing *E. coli* were not isolated from 107 samples of clams collected during the summer season.

The occurrence of ESBL-/ESBL- and AmpC-producing *E. coli* STs at the sampling sites is reported in Table 6. Variability of STs and resistance to antimicrobials were found in strains isolated from clams sampled over time. One site (sampling point 2) had the greatest variability, with isolates of different STs harboring different ESBL-producing genes (Table 6).

**Table 6:** ESBL- (ESBL-/ESBL- and AmpC-) producing *E. coli* recovered from 308 samples of clams collected from harvesting areas of the Central Adriatic between July 2018 and November 2019.

Sampling point	Sample No.	Season	Phylogroup	ST	Phenotypic Resistance	Resistance genes
SP 1	AN2	Autumn	D	7401	AMP, AZI (NS), FOT, NAL, TET, SMX, TMP (MDR)	*bla*_CTX-M-27_ *tet(A)*, *aph (3″)-Ib*, *aph(6)-Id*, *sul1*, *sul2*, *dfrA17*, *aadA5*, *mph(A)*
SP 2	AN6	Winter	A	167	AMP, FOT, TAZ, NAL, TET, FOX, CIP (MDR)	*bla*_TEM-126_/*bla*_TEM-106_*, *bla*_CMY-2_, *bla*_OXA-1_, *aac(6′)-Ib-cr*, *tet(A)*, *bla*_TEM-1B_
	AN8	Winter	A	10	AMP, AZI (NS), FOT, FEP (SSD)	*bla*_CTX-M-14_, *aac(6′)-Ib-cr*, *aac(6′)-Ib3*, *cmlA1*, *mph(A)*
	AN1	Autumn	A	398	AMP, FOT, TAZ, CHL, NAL, TET, SMX (MDR)	*bla_SHV12_*, *tetB*, *tetA*, *aadA1*, *aadA2b*, *sul3*, *cmlA1*
SP 7	AN10	Spring	D	38	AMP, FOT, TAZ (I), FEP, SMX	*bla*_CTX-M-15_, *bla*_TEM-35_
SP 21	AN13	Spring	D	1299	AMP, FOT, SMX, FOX, FEP (SSD) (MDR)	*bla*_CTX-M-1_, *sul2*
	AN3	Autumn	D	69	AMP, FOT, TMP, TET, SMX, FEP (SSD) (MDR)	*bla*_CTX-M-1_, *bla*_TEM-1B_, *qnrS1*, *tet(A)*, *aph(3″)-Ib*, *aph(6)-Id*, *aadA2b*, *sul2*, *dfrA5*, *mph(A)*
	AN4	Autumn	A	10	AMP, FOT, TAZ (I), NAL, FOX, FEP (SSD)	*bla*_CTX-M-14_, *bla*_CMY-2_, *bla*_TEM-1B_
SP 24	AN12	Spring	A	46	AMP, FOT, CHL, TMP, TET, GEN SMX, FEP (SSD) (MDR)	*bla*_CTX-M-15_, *bla*_TEM-1B_, *qnrS1*, *tet(M)*, *tet(A)*, *aph(3′)-Ia, aph(3″)-Ib*, *aph(6)-Id*, *aadA1*, *aac(3)-IId*, *sul2*, *sul3*, *cmlA1*, *dfrA14*, *dfrA12*, *lnu(F)*
SP 25	AN11	Spring	B2	131	AMP, FOT, CIP, TAZ, NAL, GEN, SMX, FOX (I), FEP (MDR)	*bla*_CTX-M-15_, *bla*_OXA-1_, *bla*_TEM-1B_, *aac(6′)-Ib-cr, aac(3)-IIa*
SP 26	AN7	Winter	F	457	AMP, FOT, CIP, TAZ (I), CHL, NAL, TMP, TET, SMX, FEP(SSD) (MDR)	*bla*_CTX-M-55_, *bla*_TEM-1B_, *tet(A)*, *aph(3′)-Ia*, *aph(3″)-Ib*, *aph(6)-Id*, *sul2*, *catA2*, *dfrA14*

*bla_TEM_ gene with a sequence identity of 99.8% to *bla*_TEM-106_ (859/861 bp) and *bla*_TEM-126_ (859/861 bp).

### *Escherichia coli* Levels and ESBL-producing *Escherichia* isolates in clams

The fecal indicator *E. coli* was quantified (MPN/100 g) in the 308 clam samples. *E. coli* numbers of clam samples were grouped into two *E. coli* contamination levels (<230 MPN/100 g and ≥ 230 MPN/100 g) and were further divided depending on the detection or not of ESBL-producing *E. coli* isolates (Table 7). ESBL-producing *E. coli* isolates were significantly more likely to be present (*p* = 0.008) among clam samples with *E. coli* > 230 MPN/100 g (4 out of 28 samples, 14% C.I.95: 5–33%) than in those with *E. coli* ≤ 230 MPN/100 g (6 out of 280 samples, 2% C.I.95: 1–5%).

**Table 7:** Presence/absence of ESBL- (ESBL- and ESBL-/AmpC-) producing *E. coli* isolates in 308 samples of clams according to the levels of *E. coli* (<230 MPN/100 g and ≥ 230 MPN/100 g).

*E. coli* MPN/100 g	No. of samples analyzed	No. of positive samples for ESBL-producing *E. coli* ^*^(%)
>230	28	4 (14%)
≤230	280	6 (2%)
Total	308	10 (3%)

The ETEC and two of the three ExPEC strains were isolated from samples with *E. coli* contamination levels >230 MPN/100 g. The majority (7 out of 11, 63%) of ESBL- and ESBL/AmpC-producing *E. coli* isolates were recovered from samples of areas classified as not suitable for direct human consumption.

## Discussion

Gut bacteria from humans or terrestrial animals, including those resistant to antimicrobials, can enter aquatic environments through various routes. Thus, bivalves, which are filter-feeder animals, may act as possible carriers of bacteria derived from fecal pollution. Antimicrobial resistance monitoring programs in the EU focus on terrestrial animals, while studies on the presence of ESBL-producing bacteria in seafood products are limited.

We report here on the presence of ESBL-producing *E. coli* strains in bivalves collected in Italy between 2018 and 2019 from sampling points of production areas of the Central Adriatic Sea. To our knowledge, this is one of the few studies performed worldwide to investigate the occurrence of ESBL-producing *E. coli* strains in bivalves from production areas (Rees et al., 2015; Bueris et al., 2022). Moreover, this study investigated the relationship between levels of *E. coli*, the bacterial indicator of fecal contamination, and the presence of *E. coli* strains with an ESBL phenotype in bivalves. Overall, *E. coli* producers of ESBL were recovered from 3% of clam samples collected at several sampling sites in the studied period; of these, 2% and 1% harbored ESBL- and ESBL-/AmpC-producing *E. coli* isolates, respectively. Previous studies in bivalve production areas were conducted in other countries and were limited in the sample collection period (Rees et al., 2015; Bueris et al., 2022). In a study conducted in Canada (Rees et al., 2015), with a study period of 2 months, ESBL-producing *E. coli* isolates were not recovered from oysters harvested from sampling points of an open oyster fishery and a restricted zone for bivalves (Rees et al., 2015). In another study from Brazil, ceftriaxone-resistant *E. coli* isolates with an ESBL phenotype were cultured from edible bivalves (oysters and brown mussels) collected from three locations of a polluted area on the South American Atlantic coast (Bueris et al., 2022). Other studies reporting on the prevalence of ESBL-producing *E. coli* in bivalves were performed at retail in European countries (Boss et al., 2016; Vu et al., 2018). In the latter studies, *E. coli* producers of ESBL or AmpC were not recovered from retail sampled oysters (*n* = 10) in Switzerland (Boss et al., 2016), whereas in Germany, ESBL-producing Enterobacteriaceae were isolated in 20% of bivalve samples collected at retail level in Berlin, with the bivalves originating from several European countries, including Italy (Vu et al., 2018). Another study from North Africa reported a prevalence of 1.6% of ESBL-producing Enterobacterales (mostly *E. coli*) among 215 analyzed pools of 5 clams (*Ruditapes decussatus*), which were sampled in unrelated markets in four different regions of Tunisia (Sola et al., 2022).

All ESBL- or ESBL-/AmpC-producing *E. coli* isolated in this study from bivalves were resistant to cefotaxime, a third-generation cephalosporin, and, to a lesser extent, to ceftazidime (27%) and fourth-generation cephalosporin cefepime (18%). Moreover, resistance to fluoroquinolones, another antibiotic class recognized as the highest priority critically important antimicrobials (HPCIAs) in human medicine (World Health Organization, 2019), was recorded in 27% of the ESBL- or ESBL-/AmpC-producing *E. coli* isolates.

Other studies have reported the proportion of third- or fourth-generation cephalosporin-resistant isolates of *E. coli* relative to the total number of isolates recovered from bivalve samples from production areas (Vignaroli et al., 2016; Grevskott et al., 2017; Miotto et al., 2019; Jeong et al., 2021). However, a screening method of a selective medium with an antibiotic to isolate third-generation cephalosporin-resistant *E. coli* strains was not applied in these studies; therefore, the prevalence of ESBL in samples collected at harvesting areas was not determined. In a systematic review and meta-analysis on antimicrobial resistance in marine bivalves from our group, resistance to third/fourth/fifth-generation cephalosporins and fluoroquinolones was recorded in approximately 10% of *E. coli* isolates, while resistance to carbapenems was not reported in *E. coli* strains from bivalves (Albini et al., 2022). Accordingly, resistance to carbapenems (ertapenem, imipenem, and meropenem) was not found in the ESBL- and ESBL-/AmpC-producing *E. coli* strains we isolated from bivalves.

CTX-M-type enzymes are the most common global ESBL in *E. coli*; among these, CTX-M-15 is the most frequent, followed by CTX-M-14 (Peirano and Pitout, 2019). Noteworthy, a recent study performed in Italy has reported CTX-M types as prevalent in both ExPEC human and animal isolates, and among these, the CTX-M-15 enzyme is largely predominant in human isolates and in a consistent percentage of the isolates from different animal species (Giufrè et al., 2021). According to the same study, the second most common CTX-M enzyme in Italian isolates was CTX-M-27 in humans and CTX-M-1 in animals. Previous studies performed on bivalve mollusks reported that the ESBL phenotype in *E. coli* was predominantly due to the presence of *bla*_CTX-M_ genes (Vu et al., 2018; Bueris et al., 2022; Sola et al., 2022), with the most frequent one detected in isolates from clams sampled at retail in Tunisia being *bla*_CTX-M-15_, followed by *bla*_CTX-M-1_ and *bla*_CTX-M-14_ (Sola et al., 2022)_._ In agreement with these studies, our analysis of ESBL genes revealed that the most prevalent gene found in *E. coli* isolates from bivalves of the Central Adriatic Sea in the studied period was the *bla*_CTX-M-15_ gene, followed by *bla*_CTX-M-14_ and *bla*_CTX-M-1_, whereas *bla*_CTX-M27_ and *bla*_CTX-M55_ were less represented. Interestingly, other ESBL-producing genes were found in a minor percentage of the *E. coli* isolates from clams (*bla*_SHV12_ and *bla*_TEM_ genes). Additionally, 18% of ESBL-producing *E. coli* isolates from clams possessed the *bla*_CMY-2_ gene for the plasmidic class C β-lactamases. Of the latter, only the CMY-2 enzyme was identified in clams. Analysis of the chromosomal or plasmid location evidenced that the majority of ESBL- /AmpC-encoding genes were harbored in sequences classified as plasmid-derived by the MOB suite. The genomic location of ESBL- /AmpC-encoding genes on plasmids in *E. coli* from the marine environment is worrying from a public health perspective as plasmids play an important role in the horizontal transfer of resistance genes. Moreover, mobile genetic elements such as insertion sequences (IS*Ecp1*, synonym of ISEc9, and IS6100) and transposons (Tn2) were also found in the same contigs of the *bla*_CTX-M_ (in five isolates) and the *bla*_CMY-2_ genes (in two isolates).

Phylogenetic group analysis showed that phylogroup A was the most prevalent (45%) in ESBL- and ESBL/AmpC-producing *E. coli* isolates, followed by other phylogroups that include isolates associated with human extra-intestinal infections (D, F, and B2). Among the sequenced ESBL- and ESBL-/AmpC-producing *E. coli* isolates, a high genomic diversity (10 different STs in 11 isolates) was observed, yet some clinically important STs were identified (ST131, ST38, ST10, ST69, ST457, and ST398). Of these, *E. coli* ST131, ST69, and ST457 showed ExPEC status and carried *bla*_CTX-M_ variants. The *E. coli* ST131 isolate from clams was MDR and had genomic features of clade C of the pandemic *E. coli* ST131 lineage (Denamur et al., 2021), which is the most prevalent ExPEC clonal group isolated in extra-intestinal infections in humans (Nicolas-Chanoine et al., 2014). The ST131 clone has previously been reported in water environments (Colomer-Lluch et al., 2013; Nicolas-Chanoine et al., 2014; Jørgensen et al., 2017), influent (Nicolas-Chanoine et al., 2014; Jørgensen et al., 2017), effluent treated wastewaters of water treatment plants (Zhi et al., 2020; Sekizuka et al., 2022), and bivalves (Vignaroli et al., 2016; Bueris et al., 2022; Sola et al., 2022). The ST457 is a broad host range, globally disseminated diverse *E. coli* lineage that can cause human extra-intestinal disease (Nesporova et al., 2021). A study focusing on ST457 evidenced that Australian human clinical and silver gull strains were closely related, suggesting that ST457 was an emerging ESBL lineage with reservoirs in wildlife and food-producing animals (Nesporova et al., 2021). ST38, ST10, and ST69 found in clams in a study conducted in Italy were reported to be more frequently detected in both human and animal isolates (Giufrè et al., 2021). Moreover, ST10 was one of the most represented STs among isolates from cattle and pigs, while ST 69 was also largely represented in isolates from pigs (Giufrè et al., 2021).

In this study, other ESBL-producing *Escherichia* spp., *E. marmotae* (Liu et al., 2015) and *E*. *ruysiae* (Van der Putten et al., 2021), were identified. Phylogroup analysis had previously assigned these species to *Escherichia* cryptic clades V and III, respectively. Previous studies on *Escherichia* cryptic clades have speculated that these may represent environmentally adapted *Escherichia* lineages that may be more abundant outside the gastrointestinal tract of the host (Walk et al., 2009; Ingle et al., 2011). Cryptic lineages of *Escherichia* were unlikely to be detected in human fecal samples and were more abundant in animal feces, ranging from 3% to 8% in non-human mammals to 8–28% in birds (Clermont et al., 2011). *E. marmotae* isolated from the feces of wild rodents (*Marmota himalayana*) has been reported as a potential invasive pathogen (Liu et al., 2019). In addition, human-invasive infections caused by *E. marmotae* have recently been described, and this *Escherichia* species has only recently been identified as a new common pathogen because it can be easily misidentified as *E. coli* in routine diagnostic laboratories (Sivertsen et al., 2022). Like animal isolates from wild rodents (Liu et al., 2019) and clinical isolates from human-invasive infections (Sivertsen et al., 2022), the ESBL-producing isolate of *E. marmotae* from clams harbored the enterotoxin-encoding gene *astA*. Moreover, the *E. coli in silico* serotyper evidenced that it contained the *fliC*-H56 flagellar antigen gene, as did the human clinical isolates (Sivertsen et al., 2022). Available data on *E. marmotae* isolates from different sources showed infrequent occurrences of antimicrobial resistance (Sivertsen et al., 2022); isolates from reported human-invasive infections were phenotypically susceptible to tested antimicrobials, and resistance genes were not identified in their genomes (Sivertsen et al., 2022). Differently from these findings, the *E. marmotae* strain from clams was an ESBL-producing isolate, phenotypically resistant to cefotaxime, which possessed the *bla*_CTX-M-1_ gene.

*Escherichia ruysiae* sp. nov. was proposed by van der Putten et al. (2021) as a novel species, encompassing *Escherichia* cryptic clades III and IV. The strain of *E*. *ruysiae* was isolated from the fecal material of an international traveler, harbored the *bla*_CTX-M-14_ gene, and was of cryptic clade IV (van der Putten et al., 2021). Differently from the human isolate, the isolate of ESBL-producing *E*. *ruysiae* from clams in this study harbored the *bla*_CTX-M-15_ gene, the enterotoxin-encoding gene *astA*, and belonged to cryptic clade III.

In the EU, the microbiological safety of bivalve mollusks is based on the classification and monitoring of production areas. Assessing the sources and types of fecal contamination in the vicinity of the areas and how these affect mollusk production areas, combined with the quantitative monitoring of the fecal indicator organism *E. coli*, is critical to providing an estimate of the risk of contamination of an area by microbial pathogens.

To the best of our knowledge, this study was the first to investigate the correlation between the presence of ESBL-producing *E. coli* and the bacterial indicator of fecal contamination of *E. coli* in bivalve mollusks. Over the studied period, ESBL (including ESBL-/AmpC-)-producing *E. coli* were not isolated in clams from most (75%) of the studied sampling sites (*n* = 28), while the highest frequency of isolation (27%) was observed in an area requiring post-harvest treatment to reduce microbiological contamination before human consumption. A significant correlation was found between the indicator of fecal contamination by *E. coli* above 230 MPN/100 g and the presence of ESBL-producing *E. coli*. Thus, this study provided evidence of *E. coli* in molluscan shellfish as an index of the potential presence of ESBL-producing *E. coli* isolates, which are bacteria resistant to a critically important class of highest-priority antimicrobials.

Considering seasonality, ESBL-producing *E. coli* were not isolated in clam samples collected in summer but in other seasons (8% in spring, 5% in winter, and 4% in autumn), although these differences in prevalence were not found to be significant. One area had the greatest variability in STs and ESBL-producing genes, whereas the *bla*_CTX-M-1_ variant was present in one specific area. These findings can be explained by the variability in the presence and type of pollution sources affecting the microbiological contamination of bivalve mollusk areas and by environmental effects (e.g., rainfalls, winds, and tidal currents) on pollution sources. A large number of uncertainties exist with respect to the sources and transmission routes of antimicrobial-resistant bacteria and antimicrobial-resistance genes in food-producing environments.

In future studies, we will investigate the presence in bivalves of ESBL-producing *E. coli*, other antimicrobial-resistant bacteria, and antimicrobial-resistance genes of importance in human health in relation to the sources and types of pollution, seasonal variations, and climatic factors that influence production areas.

In conclusion, this study presents novel observations on the prevalence, seasonality, genomic, and phenotypic characteristics of ESBL-producing *E. coli* isolates in bivalves from production areas. ESBL-producing *E. coli* isolates were significantly more likely to be present among clam samples with higher levels of *E. coli* contamination (> 230 MPN/100 g) than among those with lower levels (≤ 230 MPN/100 g). Furthermore, potentially pathogenic ESBL-producing *E. coli* strains (ETEC and ExPEC) were isolated mainly in samples with *E. coli* contamination levels above 230 MPN/100 g. These findings provided evidence in support of *E. coli* as an index organism for the presence of ESBL-producing *E. coli*.

## Data availability statement

The dataset is available in Appendix 1 of Supplementary Material. Sequencing data are published in online repositories and accession numbers are specified in the material and methods section.

## Author contributions

FL: study conception. FL, CM, FB, FM, EA, LC, and CC: methodology. FL, CM, and FB: validation and study design. LS, GA, SP, EA, and FM: laboratory analysis. FL, AR, and FM: bioinformatics analysis. LC: isolate preparation for NGS analysis. CC and SP: NGS sequencing. FB: statistical analysis. FL, FB, EA, and FM: data curation. AD and FL: resources, project administration, and funding acquisition. FL: writing–original draft preparation. CM, FB, EA, and FM: writing–reviewing and editing supervision. All authors contributed to the article and approved the submitted version.

## Footnotes

^1^STEC Reference Center, http://www.shigatox.net/new/tools/ecmlst.html.

^2^DTU, https://www.genomicepidemiology.org/services/.

^3^ http://clermontyping.iame-research.center
