# Supplementary material for: Occurrence and temporal distribution of extended-spectrum β-lactamase-producing Escherichia coli in clams from the Central Adriatic, Italy
Source: Front Microbiol. 2023 Nov 6;14:1219008. doi: 10.3389/fmicb.2023.1219008 (PMC10657901; doi:10.3389/fmicb.2023.1219008)
Supplement: Supplementary file 1 [file Data_Sheet_1.zip › Appendix 1.DOCX]

**Appendix 1**. *Escherichia coli* enumeration in MPN/100g in 308 clam samples collected in Italy from harvesting areas of the Central Adriatic between July 2018 and November 2019.

| **Sampling point** | **Date of sampling** | ***Escherichia coli* MPN/100g** | ***Escherichia coli* ESBL** |
| --- | --- | --- | --- |
| SP 17 | July 2018 | 20 | Not detected |
| SP 16 | July 2018 | 45 | Not detected |
| SP 15 | July 2018 | <18 | Not detected |
| SP 22 | July 2018 | <18 | Not detected |
| SP 23 | July 2018 | 20 | Not detected |
| SP 21 | July 2018 | 40 | Not detected |
| SP 20 | July 2018 | 20 | Not detected |
| SP 19 | July 2018 | <18 | Not detected |
| SP 18 | July 2018 | 45 | Not detected |
| SP 6 | July 2018 | 78 | Not detected |
| SP 7 | July 2018 | 78 | Not detected |
| SP 8 | July 2018 | 230 | Not detected |
| SP 10 | July 2018 | 490 | Not detected |
| SP 12 | July 2018 | 78 | Not detected |
| SP 13 | July 2018 | 20 | Not detected |
| SP 14 | July 2018 | 78 | Not detected |
| SP 11 | July 2018 | 20 | Not detected |
| SP 9 | July 2018 | 45 | Not detected |
| SP 25 | July 2018 | <18 | Not detected |
| SP 24 | July 2018 | <18 | Not detected |
| SP 15 | August 2018 | 78 | Not detected |
| SP 16 | August 2018 | 130 | Not detected |
| SP 17 | August 2018 | 78 | Not detected |
| SP 18 | August 2018 | 78 | Not detected |
| SP 19 | August 2018 | 78 | Not detected |
| SP 20 | August 2018 | 130 | Not detected |
| SP 21 | August 2018 | <18 | Not detected |
| SP 22 | August 2018 | 45 | Not detected |
| SP 23 | August 2018 | 78 | Not detected |
| SP 6 | August 2018 | <18 | Not detected |
| SP 7 | August 2018 | <18 | Not detected |
| SP 8 | August 2018 | <18 | Not detected |
| SP 10 | August 2018 | <18 | Not detected |
| SP 12 | August 2018 | <18 | Not detected |
| SP 13 | August 2018 | 45 | Not detected |
| SP 14 | August 2018 | <18 | Not detected |
| SP 11 | August 2018 | 20 | Not detected |
| SP 9 | August 2018 | <18 | Not detected |
| SP 15 | August 2018 | 20 | Not detected |
| SP 16 | August 2018 | 78 | Not detected |
| SP 17 | August 2018 | 78 | Not detected |
| SP 18 | August 2018 | 45 | Not detected |
| SP 19 | August 2018 | 790 | Not detected |
| SP 20 | August 2018 | 20 | Not detected |
| SP 21 | August 2018 | 230 | Not detected |
| SP 22 | August 2018 | 130 | Not detected |
| SP 23 | August 2018 | 20 | Not detected |
| SP 6 | September 2018 | <18 | Not detected |
| SP 7 | September 2018 | 20 | Not detected |
| SP 8 | September 2018 | 20 | Not detected |
| SP 10 | September 2018 | 78 | Not detected |
| SP 12 | September 2018 | <18 | Not detected |
| SP 13 | September 2018 | 45 | Not detected |
| SP 14 | September 2018 | 78 | Not detected |
| SP 11 | September 2018 | 20 | Not detected |
| SP 9 | September 2018 | 20 | Not detected |
| SP 17 | September 2018 | 45 | Not detected |
| SP 16 | September 2018 | 45 | Not detected |
| SP 15 | September 2018 | <18 | Not detected |
| SP 18 | September 2018 | <18 | Not detected |
| SP 19 | September 2018 | 20 | Not detected |
| SP 20 | September 2018 | <18 | Not detected |
| SP 21 | September 2018 | 130 | Not detected |
| SP 22 | September 2018 | 130 | Not detected |
| SP 23 | September 2018 | <18 | Not detected |
| SP 8 | October 2018 | 78 | Not detected |
| SP 10 | October 2018 | 170 | Not detected |
| SP 12 | October 2018 | <18 | Not detected |
| SP 13 | October 2018 | <18 | Not detected |
| SP 14 | October 2018 | <18 | Not detected |
| SP 11 | October 2018 | <18 | Not detected |
| SP 9 | October 2018 | <18 | Not detected |
| SP 7 | October 2018 | 170 | Not detected |
| SP 6 | October 2018 | 20 | Not detected |
| SP 17 | October 2018 | 68 | Not detected |
| SP 16 | October 2018 | 20 | Not detected |
| SP 15 | October 2018 | 68 | Not detected |
| SP 18 | October 2018 | <18 | Not detected |
| SP 19 | October 2018 | <18 | Not detected |
| SP 20 | October 2018 | <18 | Not detected |
| SP 21 | October 2018 | <18 | Not detected |
| SP 22 | October 2018 | 20 | Not detected |
| SP 23 | October 2018 | <18 | Not detected |
| SP 25 | October 2018 | <18 | Not detected |
| SP 24 | October 2018 | 68 | Not detected |
| SP 11 | November 2018 | <18 | Not detected |
| SP 14 | November 2018 | 20 | Not detected |
| SP 13 | November 2018 | 45 | Not detected |
| SP 12 | November 2018 | 130 | Not detected |
| SP 10 | November 2018 | 78 | Not detected |
| SP 8 | November 2018 | 45 | Not detected |
| SP 6 | November 2018 | <18 | Not detected |
| SP 7 | November 2018 | <18 | Not detected |
| SP 9 | November 2018 | <18 | Not detected |
| SP 25 | November 2018 | 330 | Not detected |
| SP 24 | November 2018 | 20 | Not detected |
| SP 17 | November 2018 | 130 | Not detected |
| SP 16 | November 2018 | 45 | Not detected |
| SP 15 | November 2018 | <18 | Not detected |
| SP 18 | November 2018 | 20 | Not detected |
| SP 19 | November 2018 | 20 | Not detected |
| SP 20 | November 2018 | <18 | Not detected |
| SP 21 | November 2018 | 20 | Not detected |
| SP 22 | November 2018 | 20 | Not detected |
| SP 23 | November 2018 | 20 | Not detected |
| SP 2 | November 2018 | 2400 | Detected |
| SP 1 | November 2018 | 9200 | Detected |
| SP 4 | November 2018 | 1700 | Not detected |
| SP 3 | November 2018 | 790 | Not detected |
| SP 14 | December 2018 | <18 | Not detected |
| SP 11 | December 2018 | <18 | Not detected |
| SP 9 | December 2018 | <18 | Not detected |
| SP 7 | December 2018 | <18 | Not detected |
| SP 6 | December 2018 | <18 | Not detected |
| SP 13 | December 2018 | <18 | Not detected |
| SP 10 | December 2018 | <18 | Not detected |
| SP 12 | December 2018 | <18 | Not detected |
| SP 8 | December 2018 | <18 | Not detected |
| SP 21 | December 2018 | 170 | Detected |
| SP 22 | December 2018 | 230 | Not detected |
| SP 17 | December 2018 | 170 | Not detected |
| SP 19 | December 2018 | 220 | Not detected |
| SP 20 | December 2018 | 78 | Not detected |
| SP 23 | December 2018 | 45 | Not detected |
| SP 16 | December 2018 | 45 | Not detected |
| SP 18 | December 2018 | 230 | Not detected |
| SP 15 | December 2018 | 170 | Not detected |
| SP 28 | December 2018 | 20 | Not detected |
| SP 27 | December 2018 | 110 | Not detected |
| SP 26 | December 2018 | 40 | Not detected |
| SP 4 | December 2018 | 68 | Not detected |
| SP 3 | December 2018 | 460 | Not detected |
| SP 1 | December 2018 | 790 | Not detected |
| SP 2 | December 2018 | 78 | Not detected |
| SP 1 | January 2019 | 18 | Not detected |
| SP 2 | January 2019 | 78 | Detected |
| SP 3 | January 2019 | 20 | Not detected |
| SP 4 | January 2019 | <18 | Not detected |
| SP 5 | January 2019 | <18 | Not detected |
| SP 26 | January 2019 | <18 | Not detected |
| SP 27 | January 2019 | 45 | Not detected |
| SP 28 | January 2019 | 40 | Not detected |
| SP 6 | February 2019 | <18 | Not detected |
| SP 7 | February 2019 | <18 | Not detected |
| SP 8 | February 2019 | <18 | Not detected |
| SP 9 | February 2019 | <18 | Not detected |
| SP 10 | February 2019 | <18 | Not detected |
| SP 11 | February 2019 | <18 | Not detected |
| SP 12 | February 2019 | <18 | Not detected |
| SP 13 | February 2019 | <18 | Not detected |
| SP 14 | February 2019 | <18 | Not detected |
| SP 28 | February 2019 | 130 | Not detected |
| SP 26 | February 2019 | 330 | Detected |
| SP 27 | February 2019 | 230 | Not detected |
| SP 1 | February 2019 | 68 | Not detected |
| SP 2 | February 2019 | 110 | Detected |
| SP 3 | February 2019 | <18 | Not detected |
| SP 4 | February 2019 | 45 | Not detected |
| SP 25 | February 2019 | <18 | Not detected |
| SP 24 | February 2019 | <18 | Not detected |
| SP 17 | February 2019 | <18 | Not detected |
| SP 16 | February 2019 | <18 | Not detected |
| SP 15 | February 2019 | <18 | Not detected |
| SP 18 | February 2019 | 20 | Not detected |
| SP 19 | February 2019 | <18 | Not detected |
| SP 20 | February 2019 | 20 | Not detected |
| SP 21 | February 2019 | 20 | Not detected |
| SP 22 | February 2019 | <18 | Not detected |
| SP 23 | February 2019 | <18 | Not detected |
| SP 2 | March 2019 | 40 | Not detected |
| SP 1 | March 2019 | 230 | Not detected |
| SP 4 | March 2019 | 20 | Not detected |
| SP 3 | March 2019 | <18 | Not detected |
| SP 6 | March 2019 | <18 | Not detected |
| SP 7 | March 2019 | 20 | Not detected |
| SP 8 | March 2019 | 20 | Not detected |
| SP 9 | March 2019 | <18 | Not detected |
| SP 10 | March 2019 | <18 | Not detected |
| SP 11 | March 2019 | 20 | Not detected |
| SP 12 | March 2019 | <18 | Not detected |
| SP 13 | March 2019 | <18 | Not detected |
| SP 14 | March 2019 | <18 | Not detected |
| SP 17 | March 2019 | <18 | Not detected |
| SP 16 | March 2019 | 20 | Not detected |
| SP 15 | March 2019 | 20 | Not detected |
| SP 18 | March 2019 | 700 | Not detected |
| SP 19 | March 2019 | 78 | Not detected |
| SP 20 | March 2019 | 110 | Not detected |
| SP 21 | March 2019 | 78 | Not detected |
| SP 22 | March 2019 | <18 | Not detected |
| SP 23 | March 2019 | 20 | Not detected |
| SP 28 | March 2019 | 20 | Not detected |
| SP 27 | March 2019 | 20 | Not detected |
| SP 26 | March 2019 | <18 | Not detected |
| SP 24 | March 2019 | <18 | Not detected |
| SP 25 | March 2019 | <18 | Not detected |
| SP 4 | April 2019 | 20 | Not detected |
| SP 2 | April 2019 | <18 | Not detected |
| SP 1 | April 2019 | <18 | Not detected |
| SP 3 | April 2019 | 20 | Not detected |
| SP 18 | April 2019 | <18 | Not detected |
| SP 17 | April 2019 | <18 | Not detected |
| SP 16 | April 2019 | <18 | Not detected |
| SP 15 | April 2019 | <18 | Not detected |
| SP 19 | April 2019 | <18 | Not detected |
| SP 20 | April 2019 | <18 | Not detected |
| SP 21 | April 2019 | <18 | Not detected |
| SP 22 | April 2019 | <18 | Not detected |
| SP 23 | April 2019 | <18 | Not detected |
| SP 10 | April 2019 | <18 | Not detected |
| SP 11 | April 2019 | <18 | Not detected |
| SP 14 | April 2019 | <18 | Not detected |
| SP 7 | April 2019 | 20 | Detected |
| SP 25 | May 2019 | 1100 | Detected |
| SP 24 | May 2019 | 170 | Detected |
| SP 17 | May 2019 | 2400 | Not detected |
| SP 16 | May 2019 | 230 | Not detected |
| SP 15 | May 2019 | 330 | Not detected |
| SP 18 | May 2019 | 170 | Not detected |
| SP 19 | May 2019 | 330 | Not detected |
| SP 20 | May 2019 | 78 | Not detected |
| SP 21 | May 2019 | 78 | Not detected |
| SP 22 | May 2019 | 1700 | Not detected |
| SP 23 | May 2019 | 490 | Not detected |
| SP 26 | May 2019 | 330 | Not detected |
| SP 27 | May 2019 | 130 | Not detected |
| SP 28 | May 2019 | 170 | Not detected |
| SP 6 | June 2019 | 790 | Not detected |
| SP 9 | June 2019 | 1300 | Not detected |
| SP 7 | June 2019 | 110 | Not detected |
| SP 8 | June 2019 | 330 | Not detected |
| SP 14 | June 2019 | 230 | Not detected |
| SP 10 | June 2019 | 78 | Not detected |
| SP 11 | June 2019 | 330 | Not detected |
| SP 12 | June 2019 | 93 | Not detected |
| SP 13 | June 2019 | 110 | Not detected |
| SP 1 | June 2019 | 18 | Not detected |
| SP 2 | June 2019 | 230 | Not detected |
| SP 3 | June 2019 | 170 | Not detected |
| SP 4 | June 2019 | 110 | Not detected |
| SP 5 | June 2019 | 20 | Not detected |
| SP 15 | June 2019 | <18 | Not detected |
| SP 19 | June 2019 | 40 | Not detected |
| SP 16 | June 2019 | 110 | Not detected |
| SP 17 | June 2019 | 1700 | Not detected |
| SP 18 | June 2019 | <18 | Not detected |
| SP 20 | June 2019 | <18 | Not detected |
| SP 21 | June 2019 | 20 | Detected |
| SP 22 | June 2019 | <18 | Not detected |
| SP 23 | June 2019 | <18 | Not detected |
| SP 5 | July 2019 | <18 | Not detected |
| SP 6 | July 2019 | <18 | Not detected |
| SP 7 | July 2019 | 20 | Not detected |
| SP 8 | July 2019 | <18 | Not detected |
| SP 9 | July 2019 | <18 | Not detected |
| SP 10 | July 2019 | 20 | Not detected |
| SP 11 | July 2019 | 45 | Not detected |
| SP 12 | July 2019 | 20 | Not detected |
| SP 13 | July 2019 | <18 | Not detected |
| SP 14 | July 2019 | 78 | Not detected |
| SP 23 | July 2019 | <18 | Not detected |
| SP 22 | July 2019 | 78 | Not detected |
| SP 20 | July 2019 | <18 | Not detected |
| SP 19 | July 2019 | 78 | Not detected |
| SP 18 | July 2019 | 130 | Not detected |
| SP 16 | July 2019 | 78 | Not detected |
| SP 17 | July 2019 | 230 | Not detected |
| SP 15 | July 2019 | 170 | Not detected |
| SP 21 | July 2019 | 45 | Not detected |
| SP 5 | July 2019 | <18 | Not detected |
| SP 2 | July 2019 | 20 | Not detected |
| SP 3 | July 2019 | <18 | Not detected |
| SP 4 | July 2019 | 45 | Not detected |
| SP 1 | July 2019 | 20 | Not detected |
| SP 27 | July 2019 | <18 | Not detected |
| SP 28 | July 2019 | <18 | Not detected |
| SP 26 | July 2019 | 20 | Not detected |
| SP 6 | July 2019 | <18 | Not detected |
| SP 7 | July 2019 | 16000 | Not detected |
| SP 8 | July 2019 | 700 | Not detected |
| SP 9 | July 2019 | 40 | Not detected |
| SP 10 | July 2019 | 230 | Not detected |
| SP 11 | July 2019 | 45 | Not detected |
| SP 12 | July 2019 | 78 | Not detected |
| SP 13 | July 2019 | 20 | Not detected |
| SP 14 | July 2019 | 220 | Not detected |
| SP 1 | August 2019 | <18 | Not detected |
| SP 2 | August 2019 | <18 | Not detected |
| SP 3 | August 2019 | <18 | Not detected |
| SP 4 | August 2019 | <18 | Not detected |
| SP 1 | September 2019 | <18 | Not detected |
| SP 2 | September 2019 | <18 | Not detected |
| SP 26 | September 2019 | 1100 | Not detected |
| SP 27 | September 2019 | 700 | Not detected |
| SP 28 | September 2019 | 490 | Not detected |
| SP 24 | October 2019 | <18 | Not detected |
| SP 5 | October 2019 | <18 | Not detected |
| SP 26 | October 2019 | 20 | Not detected |
| SP 27 | October 2019 | <18 | Not detected |
| SP 28 | October 2019 | 45 | Not detected |
| SP 4 | November 2019 | <18 | Not detected |
| SP 3 | November 2019 | 230 | Not detected |
| SP 2 | November 2019 | <18 | Not detected |
| SP 1 | November 2019 | <18 | Not detected |
| SP 5 | November 2019 | <18 | Not detected |
| SP 26 | November 2019 | 130 | Not detected |
| SP 27 | November 2019 | 210 | Not detected |
| SP 28 | November 2019 | 170 | Not detected |
