# Supplementary material for: Occurrence and temporal distribution of extended-spectrum β-lactamase-producing Escherichia coli in clams from the Central Adriatic, Italy
Source: Front Microbiol. 2023 Nov 6;14:1219008. doi: 10.3389/fmicb.2023.1219008 (PMC10657901; doi:10.3389/fmicb.2023.1219008)
Supplement: Supplementary file 1 [file Data_Sheet_1.zip › Appendix 2.PDF]

Appendix 2. Supplementary material and methods.

Sampling areas

Sampling points were chosen from north to south of the Marche region coast, which is approximately 180 km long. The 28 sampling points were distributed along the coast from north to south of the region as follows: 5 in province of Pesaro-Urbino, 18 in the province of Ancona, 2 in the province of Macerata and 3 in the province of Fermo. In the region, populated or densely populated areas are present along the coast and there are eight hydrographic basins, areas of territory identified by the presence of a basin of a main river.

Table 2.

List of antibiotics tested in the disk diffusion susceptibility tests.

**Antibiotics**	**Antibiotic abbreviation and concentration**
Ampicillin	AMP 10 µg
Cefotaxime	CTX 30 µg,
Cefoxitin	FOX 30 µg,
Chloramphenicol	C 30 µg
Ciprofloxacin	CIP 5 µg
Gentamicin	CN 10 µg
Nalidixic acid	NA 30 µg
Tetracycline	TE 30 µg
Sulfamethoxazole /trimethoprim	SXT 25 µg
