# Supplementary material for: Occurrence and temporal distribution of extended-spectrum β-lactamase-producing Escherichia coli in clams from the Central Adriatic, Italy
Source: Front Microbiol. 2023 Nov 6;14:1219008. doi: 10.3389/fmicb.2023.1219008 (PMC10657901; doi:10.3389/fmicb.2023.1219008)
Supplement: Supplementary file 1 [file Data_Sheet_1.zip › Appendix 3.DOCX]

**Appendix 3. Quality check of raw reads of *Escherichia* spp. isolates.**

| **Isolate No.** | **Bioproject No. /Accession No.** | **Total Raw Reads** | **Total Mega Base Raw** | **Q30 raw reads** | **Medium quality raw reads** | **Medium length raw reads** | **Total reads trimmed** | **Total read pairs after trimming** | **Total mega base read pairs after trimming** | **Q30 of read pairs after trimming** | **Medium quality of read pairs after trimming** | **Medium length of read pairs after trimming** |
| --- | --- | --- | --- | --- | --- | --- | --- | --- | --- | --- | --- | --- |
| AN1 | PRJNA882336 /SAMN30930934 | 2 339 576 | 348.6 | 95.02 | 34.7 | 149 | 2 285 747 | 2 237 186 | 327.37 | 97.51 | 34.77 | 146.33 |
| AN2 | PRJNA882336 /SAMN30930935 | 2 328 634 | 346.97 | 94.58 | 34.68 | 149 | 2 284 833 | 2 245 750 | 328.04 | 97.31 | 34.75 | 146.07 |
| AN3 | PRJNA882336 /SAMN30930936 | 2 204 700 | 328.5 | 94.78 | 34.68 | 149 | 2 167 265 | 2 134 256 | 312 | 97.34 | 34.74 | 146.19 |
| AN4 | PRJNA882336 /SAMN30930937 | 2 402 076 | 357.91 | 94.85 | 34.69 | 149 | 2 361 645 | 2 325 750 | 340.02 | 97.4 | 34.76 | 146.2 |
| AN5 | PRJNA882336 /SAMN30930938 | 2 312 696 | 344.59 | 95.04 | 34.65 | 149 | 2 273 255 | 2 237 504 | 327.17 | 97.44 | 34.71 | 146.22 |
| AN6 | PRJNA882336 /SAMN30930939 | 2 268 942 | 338.07 | 94.82 | 34.68 | 149 | 2 226 272 | 2 188 126 | 319.9 | 97.37 | 34.74 | 146.2 |
| AN7 | PRJNA882336 /SAMN30930940 | 1 814 660 | 270.38 | 94.98 | 34.69 | 149 | 1 785 439 | 1 759 412 | 257.34 | 97.4 | 34.75 | 146.26 |
| AN8 | PRJNA882336 /SAMN30930941 | 2 775 626 | 413.57 | 95.54 | 34.73 | 149 | 2 730 373 | 2 689 220 | 393.8 | 97.79 | 34.79 | 146.44 |
| AN9 | PRJNA882336 /SAMN30930942 | 2 168 512 | 323.11 | 95.07 | 34.71 | 149 | 2 132 112 | 2 099 990 | 307.25 | 97.5 | 34.77 | 146.31 |
| AN10 | PRJNA882336 /SAMN30930943 | 1 982 258 | 295.36 | 94.53 | 34.67 | 149 | 1 943 847 | 1 909 668 | 279.19 | 97.18 | 34.74 | 146.2 |
| AN11 | PRJNA882336 /SAMN30930944 | 2 713 870 | 404.37 | 95.25 | 34.72 | 149 | 2 669 195 | 2 628 994 | 384.8 | 97.57 | 34.78 | 146.37 |
| AN12 | PRJNA882336 /SAMN30930945 | 2 541 230 | 378.64 | 95.13 | 34.72 | 149 | 2 495 314 | 2 454 058 | 359.14 | 97.53 | 34.79 | 146.35 |
| AN13 | PRJNA882336 /SAMN30930946 | 2 615 040 | 389.64 | 95.14 | 34.74 | 149 | 2 569 619 | 2 528 566 | 369.87 | 97.65 | 34.8 | 146.28 |
