# Supplementary material for: Occurrence and temporal distribution of extended-spectrum β-lactamase-producing Escherichia coli in clams from the Central Adriatic, Italy
Source: Front Microbiol. 2023 Nov 6;14:1219008. doi: 10.3389/fmicb.2023.1219008 (PMC10657901; doi:10.3389/fmicb.2023.1219008)
Supplement: Supplementary file 1 [file Data_Sheet_1.zip › Appendix 4.DOCX]

**Appendix 4. Quality check of genome assemblies of *Escherichia* spp. isolates.**

| **Isolate No.** | **Bioproject No. /Accession No. of raw reads** | **Contigs**  **No.** | **Longest contig (bp)** | **Total lenght**  **(bp)** | **N50** | **N75** | **L50** | **L75** | **NS FOR 100KBP** |
| --- | --- | --- | --- | --- | --- | --- | --- | --- | --- |
| AN1 | PRJNA882336 /SAMN30930934 | 455 | 237 092 | 4 753 926 | 76 802 | 35 798 | 20 | 44 | 1.45 |
| AN2 | PRJNA882336 /SAMN30930935 | 460 | 399 828 | 5 149 028 | 219 689 | 94 468 | 9 | 18 | 3.24 |
| AN3 | PRJNA882336 /SAMN30930936 | 353 | 648 176 | 5 182 189 | 303 124 | 116 125 | 7 | 13 | 5.08 |
| AN4 | PRJNA882336 /SAMN30930937 | 814 | 232 646 | 5 619 344 | 86 330 | 40 285 | 22 | 46 | 11.83 |
| AN5 | PRJNA882336 /SAMN30930938 | 590 | 480 996 | 5 098 274 | 264 437 | 101 323 | 7 | 15 | 11.08 |
| AN6 | PRJNA882336 /SAMN30930939 | 504 | 341 982 | 5 200 187 | 120 271 | 47 544 | 13 | 30 | 3.6 |
| AN7 | PRJNA882336 /SAMN30930940 | 417 | 567 011 | 5 096 223 | 207 303 | 114 002 | 8 | 16 | 3.34 |
| AN8 | PRJNA882336 /SAMN30930941 | 550 | 408 666 | 4 960 412 | 203 891 | 96 364 | 8 | 17 | 4.13 |
| AN9 | PRJNA882336 /SAMN30930942 | 531 | 864 692 | 4 906 999 | 260 612 | 139 084 | 6 | 13 | 0.45 |
| AN10 | PRJNA882336 /SAMN30930943 | 368 | 431 277 | 5 311 835 | 166 172 | 92 651 | 10 | 20 | 1.04 |
| AN11 | PRJNA882336 /SAMN30930944 | 504 | 545 134 | 5 348 443 | 246 402 | 126 633 | 7 | 15 | 6.28 |
| AN12 | PRJNA882336 /SAMN30930945 | 561 | 286 298 | 4 887 572 | 90 570 | 38 676 | 17 | 35 | 7.04 |
| AN13 | PRJNA882336 /SAMN30930946 | 586 | 251 192 | 5 474 990 | 63 198 | 31 314 | 25 | 57 | 2.39 |
